# The Influence of Lifestyle on the Incidence of Dental Caries among 3-Year-Old Japanese Children

**DOI:** 10.3390/ijerph111212611

**Published:** 2014-12-05

**Authors:** Masako Watanabe, Da-Hong Wang, Akihiro Ijichi, Chika Shirai, Yu Zou, Masayuki Kubo, Kei Takemoto, Chie Masatomi, Keiki Ogino

**Affiliations:** 1 Department of Public Health, Graduate School of Medicine, Dentistry and Pharmaceutical Sciences, Okayama University, 2-5-1 Shikata-cho, Okayama 700-8558, Japan; E-Mails: rainyjo@gmail.com (Y.Z.); masakubo@cc.okayama-u.ac.jp (M.K.); kei_takemoto@okayama-u.ac.jp (K.T.); ma.sa.chi.n.0409@gmail.com (C.M.); kogino@md.okayama-u.ac.jp (K.O.); 2 Kobe City Public Health Center, 6-5-1 Kano-cho, Chuo-ku, Kobe 650-8570, Japan; E-Mails: akihiro_ijichi@office.city.kobe.lg.jp (A.I.); chika_shirai@office.city.kobe.lg.jp (C.S.); 3 Department of Biochemistry, Okayama University of Science, 1-1 Ridai-cho, Okayama 700-0005, Japan; E-Mail: dahong@dbc.ous.ac.jp

**Keywords:** dental caries, 3-year-olds, lifestyles, risk factors, cohort study, child’s bedtime

## Abstract

The present cohort study examined how lifestyle, household environment, and caries activity test score of Japanese children at age 1.5 years affected their dental caries incidence at age 3. Inclusion criteria were 1.5-year-old children with no dental caries. Dental examinations were performed for 33,655 children who participated in routine dental examinations at 1.5 years of age, and the exam was repeated approximately 21 months later (at age 3) at the Kobe City Public Health Center in Japan. After excluding 622 children who had caries at age 1.5 and 1831 children with missing lifestyle and household environment data in the questionnaires, the final data analysis was performed on a total of 31,202 children (16,052 boys, 15,150 girls).The multivariate logistic regression analysis indicated a strong association of the consumption of sugar-sweetened beverages/snacks, less frequent tooth brushing by the parents, lack of fluoride varnish, family history of smoking, with the risk of developing dental caries. A child’s late bedtime is also one of the major risk factors for dental caries development. Further investigation is needed to examine whether the short duration or the irregularity of the sleep-wake cycle would affect early childhood oral health and whether there is a relationship between late bedtime and late night snack intake.

## 1. Introduction

Dental caries is a multifactorial-induced infectious disease. In many countries, dental caries is a major oral health problem in children [1]. Various factors may contribute to the development of dental caries in children, such as the frequent consumption of sugar-containing snacks or beverages [2,3,4,5,6], less frequent tooth brushing habits, parental smoking [7,8,9,10], and breastfeeding (prolonged breastfeeding or falling asleep while breastfeeding, *etc.*) [11,12,13,14,15,16]. In Japan, on the basis of the Maternal and Child Health Law, dental examinations for children are performed at the age of 18 months and 3 years. According to the results of the annual National Survey on Oral Health, in the 22-year period between 1989 and 2012, the prevalence of dental caries in children aged 3 years markedly decreased from 55.8% to 19.1%. However, in comparison to the children at age 18 months, the prevalence of dental caries increased almost ten times when the children turned 3 years old [17]. To understand why there is a large difference in dental caries between children at 18 months and 3 years old is an important public health issue. The present study investigated the incidence of dental caries among children who had no caries at age 1.5. The children were followed up approximately 21 months in order to understand the association of lifestyle and household environment with early childhood dental caries.

## 2. Subjects and Methods

### 2.1. Participants

**Figure 1:**
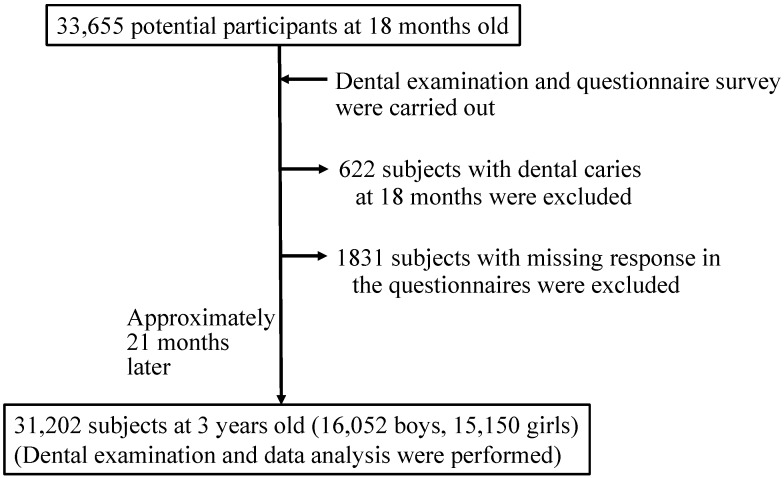
Flowchart of study cohort selection.

The subjects were recruited from the Kobe City Public Health Center in Japan between June 2006 and August 2009. As shown in Figure 1, a total of 33,655 children participated in routine dental examinations at 1.5 years old. To be included in the study, the child needed to be 1.5 years old with no dental caries. The dental examination was repeated approximately 21 months later (at age 3). After excluding 622 children who had caries at age 1.5 and 1831 children with missing responses in the lifestyle and household environment information from the questionnaires, the final data analyses were based on a total of 31,202 children (16,052 boys; 15,150 girls) who had no dental caries at 1.5 years old. These children also participated in the same routine dental examinations (21 months later at age 3) at the Kobe City Public Health Center in Japan between April 2008 and March 2011. The study was approved by the Ethics Committee of Okayama University Graduate School of Medicine, Dentistry, and Pharmaceutical Sciences. Informed consent was obtained from the guardians of all children.

### 2.2. Information on Lifestyle and Household Environment on Children Aged 1.5 Years Old

A self-administered questionnaire was completed by a parent or guardian regarding information, such as daily tooth brushing habits, varnish of fluoride, breastfeeding, consumption of sugar-sweetened beverages and snacks, child’s bedtime, and household environment, such as family smoking. The daily frequency of sweet snack intake was defined as none, once a day, twice a day, and three times or more a day. Bedtimes were classified into four groups: before 9 p.m., between 9 and 11 p.m., after 11 p.m., or irregular. Family smoking status was classified into two groups: co-residing family members who smoke or co-residing family members who do not smoke.

### 2.3. Dental Examination

The dental examination was performed in a standardized manner. Caries and tooth fillings were visually detected using an operating light and a dental mirror and a WHO/Community Periodontal Index (CPI) screening probe. The presence or absence of dental caries, including initial carious lesions, was recorded for all erupted tooth surfaces. Initial caries was defined as a demineralized surface with a chalky appearance, but without macroscopic loss of tooth substance, manifest caries was defined as the minimal level that could be verified as a cavity by probing.

### 2.4. Caries Activity Test (Cariostat)

We carried out a caries activity test for the subjects using the CAT21 test kit (Cariostat method, Willdent, Co., Osaka, Japan) to predict their future dental caries risk [18,19]. At 1.5 years old, a dental plaque sample was collected from the buccal surface of the children`s maxillary teeth with a sterile cotton swab, which was then inoculated into test medium and incubated at 37 °C for 48 h. The cariostat scores indicate the acid production by oral bacteria in the plaque through a color comparison with the 4-scale reference color patterns 0, 1.0, 2.0, 3.0 that correspond to the pH values of 6.1 ± 0.3, 5.4 ± 0.3, 4.7 ± 0.3, and 4.0 ± 0.3, respectively. A score of 0 indicates the lowest risk of dental caries, and score of 3.0 designates the highest risk of dental caries.

### 2.5. Statistical Analysis

The group comparisons were performed with the χ^2^ test for categorical variables. A multivariate logistic regression analysis was performed to determine how lifestyle and other factors contribute to the incidence of dental caries among subjects at age 3. The subjects were divided into a dichotomous dependent variable (0 = dental caries absent; 1 = dental caries present). The birth order, breastfeeding status, presence of family members who smoke, tooth brushing, child’s bedtime, varnish of fluoride, daily frequency of sweet snack intake, daily sugar-sweetened beverages consumption were defined as independent variables. The odds ratios (OR) and 95% confidence intervals (CI) were calculated. The OR was adjusted for nationality, gender, birth order, and Cariostat score. All statistical analyses were carried out using SPSS software version 16.0 for Windows (SPSS Inc., Chicago, IL, USA).

## 3. Results and Discussion

### 3.1. Characteristics of Caries-Free Subjects at Age 1.5 Years

Table 1 shows the characteristics of subjects who had no dental caries at 1.5 years of age. The proportion of breastfed subjects was 21.3% in boys and 22.6% in girls. Almost half of the children consumed sweet snacks once per day, and the other half of the children consumed sweet snacks twice or more per day. The proportion of subjects who consumed sugar-sweetened beverages was similar in boys (44.2%) and girls (43.3%). Less than one-fourth of the children (22.7%) went to bed before 9 p.m., most of the children went to bed after 9 p.m. Two-third of the boys (61.9%) and girls (64.7%) reported daily tooth brushing habits by the parents, and 86.5% subjects received fluoride varnish.

Regarding the household environment, nearly half of the co-residing family members were smokers (44.8%). In addition, more than half of the boys (55.5%) and girls (54.7%) had a Cariostat score of 1.0, and one-third of the boys (37.3%) and girls (37.6%) had a Cariostat score of 2.0. The proportion of subjects with the highest Cariostat score was similar between boys (2.5%) and girls (2.2%).

**Table 1:** Characteristics of caries-free subjects aged 1.5 years.

Variable	All (n = 31,202)	Boy (n = 16,052)	Girl (n = 15,150)
	n (%)	n (%)	n (%)
Breastfeeding	6834 (21.9)	3416 (21.3)	3418 (22.6)
Daily frequency of sweet snack intake			
0	764 (2.4)	423 (2.6)	341 (2.3)
1	15,270 (48.9)	7843 (48.9)	7427 (49.0)
2	13,529 (43.4)	6944 (43.3)	6585 (43.5)
3	1639 (5.3)	842 (5.2)	797 (5.3)
Daily sugar sweetened-beverage consumption	13,661 (43.8)	7097 (44.2)	6564 (43.3)
Child’s bedtime			
Before 9 p.m.	7081 (22.7)	3732 (23.2)	3349 (22.1)
9~11 p.m.	19,580 (62.8)	10,082 (62.8)	9498 (62.7)
After 11 p.m.	1921 (6.2)	914 (5.7)	1007 (6.6)
Irregular	2620 (8.4)	1324 (8.2)	1296 (8.6)
Daily toothbrushing by parents	19,733 (63.2)	9936 (61.9)	9797 (64.7)
Varnish of fluoride	26,990 (86.5)	13,886 (86.5)	13,104 (86.5)
Smoking of family members	13,994 (44.8)	7199 (44.8)	6795 (44.9)
Order of birth			
1st	15,323 (49.1)	7945 (49.5)	7378 (48.7)
2nd	11,981 (38.4)	6084 (37.9)	5897 (38.9)
3rd or more	3898 (12.5)	2023 (12.6)	1875 (12.4)
Cariostat score			
0.0	1583 (5.1)	759 (4.7)	824 (5.4)
1.0	17,195 (55.1)	8905 (55.5)	8290 (54.7)
2.0	11,693 (37.5)	5990 (37.3)	5703 (37.6)
3.0	731 (2.3)	398 (2.5)	333 (2.2)

### 3.2. Incidence of Dental Caries in 3-Year-Old Subjects

Table 2 shows the incidence of dental caries in 3-year-old subjects who were free from dental caries at 1.5 years old. The incidence of dental caries was significantly different between boys (17.3%) and girls (15.4%). The incidence of dental caries was 24.6% in subjects who were breastfeeding and 14.2% in subjects who did not breastfeed.

**Table 2:** Incidence of dental caries in 3-years old subjects.

Variables	All subjects (n = 31,202)		Boy (n = 16,052)		Girl (n = 15,150)	
	n (%)	*p* ^a^	n (%)	*p* ^a^	n (%)	*p* ^a^
Incidence of dental caries	5106 (16.4)		2777 (17.3)		2329 (15.4)	<0.001
Breastfeeding		<0.001		<0.001		<0.001
No	3422 (14.2)		1856 (14.7)		1566 (13.3)	
Yes	1684 (24.6)		921 (27.0)		763 (22.3)	
Daily frequency of sweet snack intake		<0.001		<0.001		<0.001
0	43 (5.6)		22 (5.2)		21 (6.2)	
1	1925 (12.6)		1054 (13.4)		871 (11.7)	
2	2733 (20.2)		1482 (21.3)		1251 (19.0)	
3	405 (24.7)		219 (26.0)		186 (23.3)	
Daily sugar-sweetened beverage consumption		<0.001		<0.001		<0.001
No	2324 (13.2)		1254 (14.0)		1070 (12.5)	
Yes	2782 (20.4)		1523 (21.5)		1259 (19.2)	
Child’s bedtime		<0.001		<0.001		<0.001
Before 9 p.m.	852 (12.0)		480 (12.9)		372 (11.1)	
9~11 p.m.	3256 (16.6)		1789 (17.7)		1467 (15.4)	
After 11 p.m.	430 (22.4)		195 (21.3)		235 (23.3)	
Irregular	568 (21.7)		313 (23.6)		255 (19.7)	
Daily toothbrushing by parents		<0.001		<0.001		<0.001
Yes	2708 (13.7)		1439 (14.5)		1269 (13.0)	
No	2398 (20.9)		1338 (21.9)		1060 (19.8)	
Varnish of fluoride		<0.001		<0.001		<0.001
Yes	4130 (15.3)		2240 (16.1)		1890 (14.4)	
No	976 (23.2)		537 (24.8)		439 (21.5)	
Smoking of family members		<0.001		<0.001		<0.001
No	2317 (13.5)		1266	(14.3)	1051 (12.6)	
Yes	2789 (19.9)		1511	(21.0)	1278 (18.8)	
Order of birth		<0.001		<0.001		<0.001
1st	1933 (12.6)		1085 (13.7)		848 (11.5)	
2nd	2121 (17.7)		1125 (18.5)		996 (16.9)	
3rd or more	1052 (27.0)		567 (28.0)		485 (25.4)	
Cariostat score		<0.001		<0.001		<0.001
0.0	155 (9.8)		79 (10.4)		76 (9.2)	
1.0	2347 (13.6)		1288 (14.5)		1059 (12.8)	
2.0	2339 (20.0)		1259 (21.0)		1080 (18.9)	
3.0	265 (36.3)		151 (37.9)		114 (34.2)	

^a^ χ^2^ test was used for group comparison.

The incidence of caries was significantly higher in children who consumed sugar-sweetened beverages daily (20.4%) than those who did not (13.2%). It is worth noting that children who went to bed before 9 pm had the lowest incidence of caries (boys: 12.9%; girls: 11.1%). Children with later bedtimes had higher incidences of caries in both boys and girls. The highest incidence of caries occurred among children who went to bed after 11 p.m.

Children who did not receive tooth brushing from their parents had a higher proportion of dental caries at 3 years old (20.9%) compared to children who received tooth brushing by their parents (13.7%). In addition, children who did not receive fluoride varnish had a higher incidence of caries (23.1%), especially in boys (24.8%) (girls 21.5%). Children living with family members who smoked had a higher incidence of dental caries (19.9%) than children living with family members who did not smoke (13.5%). There was a higher caries incidence among children who had the highest dental caries activity score (3.0) at 1.5 years old (37.9% boys; 34.2% girls).

### 3.3. Breastfeeding Was Positively Associated with Dental Caries in Children

After controlling for nationality, birth order, and Cariostat score, we found that breast-fed children had an approximately 2-fold increased risk of dental caries in both boys and girls (Table 3). In the literature, there have been conflicting results on the association between breastfeeding and caries development [4,11,12,13,14,15,20]. In a cross-sectional study with a sample of 504 Iranian children, breastfeeding (and even prolonged breastfeeding) had no negative dental consequences [21]; Iida [20] also found that breastfeeding and its duration were not associated with a risk for early childhood caries in the United States (n = 1576). However, a longitudinal study in Japan demonstrated that prolonged breastfeeding is a risk factor for dental caries [13]. In a study of American preschool children (n = 1206), children who continued breastfeeding after falling asleep had higher incidence of caries than children who did not [4]. Therefore, the evidence on whether breastfeeding increases the risk of caries is inconclusive and should be further examined.

### 3.4. Consumption of Sweet Snacks and Sugar-Sweetened Beverages Increased the Risk of Developing Dental Caries in Children

In the adjusted multivariate logistic regression analysis, children who consumed 3 times sweet snacks per day increased their risk of developing dental caries by 3.9-fold (OR: 3.90; 95% CI: 2.79, 5.45) (4.6-fold in boys; 3.2-fold in girls (Table 3)) compared with children who did not consume sweet snacks at the age of 1.5 years. Johansson *et al.* also reported that the number of sweet items used for daily snacking were independently associated with the incidence of caries [4].

**Table 3:** Multivariable logistic regression analysis of factors predicting dental caries in children at 3 years old.

Variables	All (n = 31,202)		Boy (n = 16,052)		Girl (n = 15,150)	
	OR ^a^ (95% CI ^b^)	*p*	OR ^c^ (95% CI ^b^)	*p*	OR ^c^ (95% CI ^b^)	*p*
Breastfeeding						
No	1.0		1.0		1.0	
Yes	2.06 (1.93, 2.21)	<0.001	2.21 (2.01, 2.43)	<0.001	1.91 (1.73, 2.11)	<0.001
Daily frequency of sweet snack intake						
0	1.0		1.0		1.0	
1	2.00 (1.46, 2.74)	<0.001	2.35 (1.51, 3.65)	<0.001	1.65 (1.05, 2.60)	<0.001
2	3.21 (2.34, 4.40)	<0.001	3.78 (2.43, 5.87)	<0.001	2.64 (1.68, 4.16)	<0.05
3	3.90 (2.79, 5.45)	<0.001	4.61 (2.90, 7.35)	<0.001	3.20 (1.97, 5.17)	<0.001
Daily sugar-sweetened beverage consumption						
No	1.0		1.0		1.0	
Yes	1.56 (1.46, 1.65)	<0.001	1.55 (1.42, 1.69)	<0.001	1.55 (1.41, 1.70)	<0.001
Child’s bedtime						
Before 9 p.m.	1.0		1.0		1.0	
9~11 p.m.	1.33 (1.23, 1.45)	<0.001	1.33 (1.19, 1.49)	<0.001	1.34 (1.18, 1.51)	<0.001
After 11 p.m.	1.85 (1.61, 2.12)	<0.001	1.59 (1.30, 1.93)	<0.001	2.13 (1.75, 2.58)	<0.001
Irregular	1.71 (1.51, 1.93)	<0.001	1.74 (1.47, 2.06)	<0.001	1.67 (1.39, 2.01)	<0.001
Daily toothbrushing by parents						
Yes	1.0		1.0		1.0	
No	1.32 (1.24, 1.41)	<0.001	1.30 (1.19, 1.42)	<0.001	1.35 (1.23, 1.49)	<0.001
Varnish of fluoride						
Yes	1.0		1.0		1.0	
No	1.49 (1.37, 1.62)	<0.001	1.53 (1.37, 1.72)	<0.001	1.44 (1.27, 1.63)	<0.001
Smoking of family members						
No	1.0		1.0		1.0	
Yes	1.43 (1.34, 1.52)	<0.001	1.41 (1.29, 1.54)	<0.001	1.45 (1.32, 1.59)	<0.001

^a^ OR: Odds ratio (adjusted for nationality, gender, order of birth, cariostat score); ^b^ CI: Confidence interval; ^c^ Odds ratio adjusted for nationality, order of birth, cariostat score.

In addition, the OR of caries 1.56 (95% CI: 1.46, 1.65; *p* < 0.001) was higher in children who consumed sugar-sweetened beverages daily at the age of 1.5 years compared with children who did not, and the tendencies were similar between boys and girls (Table 3). In the literature, the evidence has consistently shown an association between the consumption of sugar-containing snacks or beverages and dental caries in children [2,3,4,5,6,21].

It is well known that a high amount of simple carbohydrate consumption, such as sucrose and glucose, can increase the incidence of dental caries because cariogenic bacteria require these sugars for food. Additionally, complex carbohydrates (food starches) easily become stuck between the teeth and gums and are also considered as possible risk factors for caries by “promoting the retention of sugar on the teeth” [22]. Firestone *et al*. reported the possibility of “a caries-enhancing interaction” between complex carbohydrate starches and simple carbohydrate sugar in animal studies [23]; these findings could be supported by our results. The OR of caries for consumption of sweet snacks (OR: 2.0, 3.90) was higher than that of sugar-sweetened beverages (1.56) (Table 3) suggesting that sweet snacks may be a more important determinant of early childhood caries.

### 3.5. Late Bedtime Contributes to the Development of Dental Caries

Table 3 shows that the risk of dental caries in children at age 3 increased when their bedtime was later than 9 p.m. or irregular. In comparison with children who went to bed before 9 p.m., the odds ratios for the risk of caries development in children who went bed between 9 and 11 p.m. and after 11 p.m. are 1.33 (95% CI: 1.23, 1.45) and 1.85 (95% CI: 1.61, 2.12), respectively, according to a multivariate logistic regression analysis. This result suggests that a late bedtime is an independent risk factor for caries. One explanation for this result may be that children who go to bed late might increase their consumption of snacks around bedtime. Unfortunately, we did not have this information for the present subjects. There have been few studies regarding the association between a child’s bedtime and dental caries. Mattila *et al.* reported that a late bedtime was associated with increased caries in 10-year-old Finnish children [21]. Whether the short duration or the irregularity of the sleep-wake cycle affects early childhood oral health or whether there is a relationship between late bedtime and late snack intake should be clarified.

### 3.6. Daily Tooth Brushing Decreased Dental Caries in Children

Our results indicate that daily tooth brushing by the parents decreased the risk of caries development. Children without daily tooth brushing had a higher caries incidence (20.9% *vs.* 13.7%) (Table 2) and an elevated risk of caries development (OR: 1.32; 95% CI: 1.24, 1.41) in both boys (OR: 1.30; 95% CI: 1.19, 1.42) and girls (OR: 1.35; 95% CI: 1.23, 1.49) (Table 3). Previous studies have reported that tooth brushing and supervised tooth brushing are effective approaches to caries prevention [24,25]. However, some studies have demonstrated that tooth brushing has a limited effect in reducing the incidence of caries [26] because a toothbrush cannot remove the plaque between teeth or inside the fissures on chewing surfaces.

### 3.7. Fluoride Varnish

The present results indicate that the application of fluoride varnish at 1.5 years old is an independent variable for decreasing the risk of caries development at 3 years old (Table 3). Children who did not receive fluoride varnish had an elevated risk of caries development (OR: 1.49; 95% CI: 1.37, 1.62) in both boys (OR: 1.53; 95% CI: 1.37, 1.72) and girls (OR: 1.44; 95% CI: 1.27, 1.63). This finding is supported by existing literature [18,27,28,29]. The use of fluoride has been recommended for the prevention of dental caries by the Centers for Disease Control and Prevention [21,30]. Fluoride not only inhibits the demineralization of enamel but also remineralizes enamel crystals [30]. Furthermore, in an *in vitro* study, the presence of fluoride decreased the acid production of cariogenic bacteria [31,32]. In a large study of Australian children [6], exposure to fluoridated water decreased the association between the consumption of sugar-sweetened beverages and dental decay.

### 3.8. Household Smoking Is a Risk Factor for Development of Early Childhood Caries

Childhood caries were more frequent in children who lived with family members who smoked than children who lived with family members who did not smoke, and the OR of caries for children living with family members who smoked was 1.49 (95% CI: 1.34, 1.52) (Table 2 and Table 3). These results agreed with the results reported by Hanioka [9], who also found that the OR of caries for Japanese children with parents who smoked was 1.52 (95% CI: 1.01, 2.30). A recent systematic review has confirmed the association of secondhand smoke with early childhood caries [7]. In an *in vitro* study, environmental tobacco smoke exposure increased the growth of cariogenic bacteria (*Mutans streptococcus*) that can convert carbohydrates into acids [33]. Kohler [34] found that early colonization by *Mutans* streptococci lead to higher caries prevalence in children at age 4.

### 3.9. Main Strengths and Limitations of the Study

An important strength of the present study is that it is the first one we are aware of that has addressed the relationship between sleep habit and risk of developing dental caries in the deciduous teeth of children although Mattila *et al.* reported a child’s late bedtime was an explanatory factor in the permanent teeth [21]. The other strengths are the longitudinal study design and the large sample size.

The present study had a number of identified limitations. (1) The data were collected from one city; therefore, caution should be used when generalizing the present findings to other regions. (2) The lifestyle data, such as tooth brushing, smoking status of family members and child’s bedtime, relied on self-reporting, which might be subject to bias. (3) The dental caries severity in adults is positively associated with household income [35]. However, Tanaka [36] recently reported no relationship between household income and the risk of dental caries in preschool children. In our study, data on socioeconomic status, such as annual household income and parent’s education level, were not available.

## 4. Conclusions

The present study examined how lifestyle, household environment, and caries activity test score of Japanese children at age 1.5 affected their dental caries incidence at age 3. The findings support a positive association of dental caries incidence with the following factors: consumption of sugar-sweetened beverages and snacks, living with family members who smoke, and also suggest late bedtime is one of the major risk factors for dental caries in children. Further investigation is needed to examine whether the short duration or the irregularity of the sleep-wake cycle affects early childhood oral health and whether there is a relationship between a late bedtime and late snack intake.
